# Diacylglycerol Acetyltransferase Gene Isolated from *Euonymus europaeus* L. Altered Lipid Metabolism in Transgenic Plant towards the Production of Acetylated Triacylglycerols

**DOI:** 10.3390/life10090205

**Published:** 2020-09-16

**Authors:** Daniel Mihálik, Andrea Lančaričová, Michaela Mrkvová, Šarlota Kaňuková, Jana Moravčíková, Miroslav Glasa, Zdeno Šubr, Lukáš Predajňa, Richard Hančinský, Simona Grešíková, Michaela Havrlentová, Pavol Hauptvogel, Ján Kraic

**Affiliations:** 1 Research Institute of Plant Production, National Agricultural and Food Center, Bratislavská cesta 122, 92168 Piešt’any, Slovakia; daniel.mihalik@nppc.sk (D.M.); andrea.lancaricova@nppc.sk (A.L.); michaela.havrlentova@nppc.sk (M.H.); pavol.hauptvogel@nppc.sk (P.H.); 2 Faculty of Natural Sciences, University of Ss. Cyril and Methodius, Námestie J. Herdu 2, 91701 Trnava, Slovakia; michaela.mrkvova@ucm.sk (M.M.); sarlota.kanukova@gmail.com (Š.K.); jana.moravcikova@ucm.sk (J.M.); miroslav.glasa@ucm.sk (M.G.); rhancinsky@gmail.com (R.H.); simonagresikova@gmail.com (S.G.); 3 Institute of Virology, Biomedical Research Centre, Slovak Academy of Sciences, Dúbravská cesta 9, 84505 Bratislava, Slovakia; Zdeno.Subr@savba.sk (Z.Š.); Lukas.Predajna@savba.sk (L.P.)

**Keywords:** *Euonymus europaeus*, DGAT, DAcT, acetylTAG

## Abstract

*Euonymus* species from the Celastraceae family are considered as a source of unusual genes modifying the oil content and fatty acid composition of vegetable oils. Due to the possession of genes encoding enzyme diacylglycerol acetyltransferase (DAcT), *Euonymus* plants can synthesize and accumulate acetylated triacyglycerols. The gene from *Euonymus europaeus* (*EeDAcT*) encoding the DAcT was identified, isolated, characterized, and modified for cloning and genetic transformation of plants. This gene has a unique nucleotide sequence and amino acid composition, different from orthologous genes from other *Euonymus* species. Nucleotide sequence of original *EeDAcT* gene was modified, cloned into transformation vector, and introduced into tobacco plants. Overexpression of *EeDAcT* gene was confirmed, and transgenic host plants produced and accumulated acetylated triacylglycerols (TAGs) in immature seeds. Individual transgenic plants showed difference in amounts of synthesized acetylTAGs and also in fatty acid composition of acetylTAGs.

## 1. Introduction

*Euonymus* is a cosmopolitan genus of the family Celastraceae containing more than two hundred of species [1] mostly native in East Asia. Species *Euonymus alatus* L. is widely distributed and traditionally used as a medicinal plant in many Asian countries. More than 230 chemical compounds have been identified and isolated from it, including sesquiterpenoids, diterpenoids, triterpenoids, flavonoids, phenylpropanoids, lignans, steroids, alkaloids, and other compounds [1,2]. Generally, *Euonymus* species have the potential for treatment of many injuries, inflammation, and oxidative stress as well as diseases including cancer, diabetes, and others [2,3]. *Euonymus europaeus* L. (spindle tree, European spindle) is distributed in temperate climates from Central to Eastern Europe. It is mainly considered as an ornamental shrub and its homogenous wood is easy to work for special products. Bark, leaves, and seeds contain compounds of medicinal and veterinary value [4]. However, spindle tree is not grown as a farm plant.

An interesting and not common trait of plants from the family Celastraceae is the constitution of oils in their fruits containing seeds surrounded by arils. Oil contains unique storage lipid molecules of 3-acetyl-1,2-diacyl-*sn*-glycerols (acetylTAGs) in an amount of more than 90% of all triacylglycerols (TAGs) [5]. An acetyl group at the *sn*-3 position complements two residues of saturated or unsaturated C16–C18 fatty acids on the glycerol molecule. Introduction of an acetyl group substantially changes the properties of lipid molecules and parameters of oil. Such oil is attractive due to significantly improved parameters important in the production of biodiesel (especially reduced viscosity, freezing at lower temperature, unnecessary esterification), emulsifiers, lubricants, and plasticizers as well as in food production and human nutrition (lower caloric content, positively affects the gut microbiota) [6,7,8].

There are also interesting variations in the composition of fatty acids in TAGs and acetylTAGs in fruits present among different *Euonymus* species. The most studied species is *E. alatus* L., but *E. europaeus* L. is one of the few occurring naturally in Europe. Its dominant fatty acid in mature seeds is the monounsaturated oleic acid (C18:1), in contrast to *E. alatus* L. where the linoleic acid (C18:2) is dominant [9]. The ratio of oleic acid to linoleic acid at the *sn*-1 and *sn*-2 positions of 3-acetyl-1,2-diacyl-*sn*-glycerols in mature seeds separates *E. europaeus* and *E. alatus* into different subgenus of the *Euonymus* L. [10]. A vegetable oil with a high content of acetylTAGs and higher proportion of monounsaturated oleic acid would be very advantageous for the production of biodiesel when compared with oils with a high content of polyunsaturated fatty acids [11,12]. However, none of the vegetable oils has such a composition of fatty acids, TAGs, and acetylTAGs that would make all the biodiesel parameters optimal. Synthetic biology, genetic engineering, and transgenesis could be the solution for the creation of a commercial oil crop that would produce an ideal vegetable oil with a high proportion of desired fatty acids (C18:1, C16:1), moreover, stored in the form of acetylTAGs. The cultivation of such a crop should eliminate many current social, economic, and technical consequences associated with biodiesel production [13]. The design of oil biosynthesis in plants, that would be optimal for biodiesel production or other applications, could also be achieved through the engineering of acyltransferases and *sn*-1,2-diacylglycerol:acyl-CoA acyltransferases (DGATs) synthesizing the final TAGs and regulating their content and composition in the plant [14,15]. A novel type of DGAT enzyme has been identified in seeds of *E. alatus* L. This enzyme was named *Euonymus alatus* diacylglycerol acetyltransferase (*Ea*DAcT) [16] and it accumulates a high level of 3-acetyl-1,2-diacyl-*sn*-glycerols (acetylTAGs) as their major storage lipids [17]. The gene encoding *Ea*DAcT would be very interesting for its functional introduction into commercial oil crops. *Euonymus* itself is not suitable for the large-scale production of vegetable oil with acetylated TAGs. The expression of *EaDAcT* gene controlled by seed-specific promoter really resulted in the production of acetylTAGs in the seed oil of transgenic *Arabidopsis thaliana* [16]. Later, camelina (*Camelina sativa* L. Crantz.) and soybean (*Glycine max* L.) were transformed with the same *EaDAcT* gene, and transgenic plants accumulated acetylTAGs at up to 70 mol% of seed oil [6]. Genes encoding DGATs from another *Euonymus* species could provide a perspective for biotechnological production of acetylTAGs in transgenic organisms. This has been demonstrated with genes *Ef*DAcT, *Et*DAcT, *Eb*DAcT, and *Ek*DAcT isolated from *E*. *fortune*, *E*. *atropurpureus*, *E*. *maackii*, and *E*. *kiautschovicus* and expressed in transgenic yeast *S. cerevisiae* [18].

The *EeDAcT* gene could be used in genetic engineering of plants, especially oil crops, in designing of plant oils tailored for biofuel production as well as for nutritional applications. Therefore, aims of this study were to (i) isolate and characterize the natural gene encoding diacylglycerol acetyltransferase from *Euonymus europaeus* L. (*Ee*DAcT), (ii) modify the nucleotide sequence of isolated natural *EeDAcT* gene to be suitable for cloning, (iii) transfer modified *EeDAcT* gene into the host plants and confirm the ability of modified *EeDAcT* gene to affect the lipid synthesis and to produce acetylated triacylglycerols. 

## 2. Materials and Methods 

### 2.1. Plant Material

Seeds of *Euonymus europaeus* L. used for isolation of *DAcT* gene, subsequent synthetic gene synthesis, cloning, and plant transformation were collected at the place of its natural occurrence in locality Hrachovište (Slovakia). Tobacco plants (*Nicotiana tabacum* L.), cv. Petit Havana SR1, were cultivated from seeds obtained from the Gene Bank of the Slovak Republic (Research Institute of Plant Production, Piešťany, Slovakia).

### 2.2. Lipid Analysis

Deep-frozen plant material was mixed with a solution of chloroform and methanol (2:1, *v*/*v*) according to [19] Folch et al. (1957). The mixture was filtered through a paper filter into an Erlenmeyer flask, and potassium chloride (0.97%) was added. After gentle shaking, the mixture was centrifuged (1000× *g*, 5 min) to separate into two layers. The lower layer was collected, and solvents were evaporated under vacuum at 40 °C. The resultant oil was flushed with nitrogen and stored at −20 °C until further analysis. Yield of extracted oil was calculated.

Extracted lipids were dissolved in chloroform and applied on the thin-layer chromatography (TLC) silica gel 60 plates (Merck KGaA, Darmstadt, Germany). Plates were developed in a hexane:ether:acetic acid (80:20:1) system, visualized by iodine vapors [20], and scanned and evaluated using the UN-SCAN-IT 6.0 Graph Digitizing Software, Version 6.0 (Silk Scientific, Inc., Orem, UT, USA) [21].

Spots of lc-TAGs (long-chain TAGs) and acetyl-TAGs were scraped off the TLC plates into test tubes and extracted for methylation and gas chromatography (GC) measurements according to [22]. Fatty acids methyl esters were analyzed by gas chromatography with mass spectrometry (GC-MS) using the Agilent 7890B/5977A Series GC/MSD System (Agilent Technologies, Santa Clara, CA, USA) under the following conditions: column temperature program—initial temperature 150 °C, 4 min, then increased at 3 °C/min to 230 °C (held for 5 min), and finally increased at 15 °C/min to 280 °C (maintained 19 min). Inlet parameters (capillary column inlet): temperature 250 °C, pressure 10.8 psi, total helium pressure 169.32 mL/min, splitless. HP-5ms ultra inert column: dimensions 30 m × 250 μm × 0.25 μm, initial temperature 150 °C, pressure 10.8 psi, flow rate 0.82746 mL/min, average value 34,613 cm/s. The injection volume of the sample was 4 µL.

MS parameters: MSD transfer line temperature 280 °C, ion source temperature 230 °C, quadrupole temperature 150 °C, electron energy 70 eV, record full mass spectra (SCAN type), gain factor 1, scanning range 50–550 m/z, scan speed 1.562 (N = 2). Fatty acid identification was performed by comparing the mass spectra of samples with the spectra in the NIST 2007 library databases. The chromatograms were evaluated based on the area of peaks. The sums of saturated (SFA), mono- (MUFA) and poly-unsaturated (PUFA) fatty acids were calculated from fatty acid values.

### 2.3. EeDAcT Gene Isolation, Sequencing, and Modification

The plant genomic DNA from *E. europaeus* was extracted from 1 g of fresh leaves using the NucleoSpin^®^ Plant II kit (Macherey-Nagel GmbH & Co. KG, Dueren, Germany). Primers derived from 1,2-diacyl-*sn*-glycerol:acetyl-CoA acetyltransferase (DAcT) gene of *E. alatus* L. (GenBank accession no. GU594061.1) were used for amplification of the homologous gene *EeDAcT* from *E. europaeus* L. The complete DNA sequence of *EeDAcT* gene was amplified using forward and reverse primers 5′-AATCAAGCGAAACCCATCAC-3′ and 5′-ATCACAAACCCCATCACCAT-3′. Expected size of the PCR product was 1092 bp. The PCR reaction mixture (25 µL) contained 1 × PCR buffer, 1.5 mM MgCl_2_, 10 pM of both primers, 0.2 mM each of dNTP and 0.5 U Platinum^TM^ *Taq* DNA polymerase (Invitrogen Corp., Carlsbad, CA, USA), and 100 ng of genomic template DNA from *E. europaeus*. The PCR was performed in Mastercycler^®^ ep (Eppendorf, Hamburg, Germany) using following parameters: denaturation at 94 °C for 3 min, followed by 30 cycles, each consisting of 94 °C for 1 min, 58 °C for 30 s, 72 °C for 1 min, and the final extension at 72 °C for 10 min. 

The total RNA from *E. europaeus* was extracted from 1 g of plant tissues using the TRIzol reagent method (Invitrogen Corp., Carlsbad, CA, USA) from root, stem, leaf, aril, pericarp, and immature seed. Potential residues of genomic DNA were removed by DNase treatment (Fermentas, St. Leon-Rot, Germany). Concentration of isolated RNA was determined spectrophotometrically (Nanodrop 1000 Spectrophotometer, Thermo Fisher Scientific, Waltham, MA, USA). Quality of RNA has been verified by electrophoresis in 1.5% agarose–formaldehyde gel stained with ethidium bromide. The RevertAid First Strand cDNA Synthesis Kit (Fermentas, St. Leon-Rot, Germany) was used for the first-strand cDNA synthesis. Fifty nanograms of the first cDNA strands was used as templates for the second-strand synthesis by PCR at the same composition and cycling parameters as for genomic DNA. PCR primers were designed by the SnapGene software (GSL Biotech LLC, San Diego, CA, USA) (Table 1). Completing of *EeDAcT* gene sequence was performed by overlapping of cDNA fragment sequences obtained by amplifications with primers F1–F4 (Table 1). The PCR reaction mixture (25 µL) contained 1 × PCR buffer, 1.5 mM MgCl_2_, 10 pM of both primers, 0.2 mM each of dNTP and 0.5 U Platinum^TM^ *Taq* DNA polymerase (Invitrogen Corp., Carlsbad, CA, USA), and 100 ng of template cDNA. The PCR was performed in the same thermocycler using initial denaturation at 94 °C for 3 min, followed by 35 cycles, each of denaturation at 94 °C for 1 min, annealing at 58 °C for 1 min, extension at 72 °C for 1 min, and the final extension at 72 °C for 10 min. Products of PCR were electrophoretically analyzed in 1.5% (*w*/*v*) agarose gels pre-stained with ethidium bromide and extracted from gel using the Agarose Gel Extraction Kit (Roche Diagnostics GmbH, Mannheim, Germany). DNA sequencing was done by Sanger method in commercial sequencing service (Comenius University, Bratislava, Slovakia). Complete cDNA of the 1,2-diacyl-*sn*-glycerol:acetyl-CoA acetyltransferase from *E. europaeus* (*EeDAcT*) was submitted to the GenBank^®^ nucleotide database [23] (as accession no. MK637625.1).

Sequence alignments of cDNA and proteins of the gene *DAcT* within the family Celastraceae Celestraceae were performed using the CLC Main Workbench 20.0 software (Qiagen N.V., Venlo, The Netherlands). The phylogenetic tree was constructed using the Clustal W software [24,25]. Compared were DNA and protein sequences, respectively, from *E. europaeus* (MK637625.1, QDH76310.1), *E. maackii* (MF061250.1, ASM61114.1), *E. kiautschovicus* (MF061251.1, ASM61115.1), *E. atropurpureus* (MF061249.1, ASM61113.1), *E. alatus* (GU594061.1, ADF57327.1), *E. fortune* (MF061252.1, ASM61116.1), and *Celastrus scandens* (MF061248.1, ASM61112.1).

### 2.4. EeDAcT Gene Synthesis and Expression in Tobacco

The gene encoding the enzyme *Ee*DAcT was synthesized (Eurofins Genomics Germany GmbH, Ebersberg, Germany) in full length. The synthetic *EeDAcT* gene was modified by addition of restriction endonuclease sites compatible with cloning the gene into binary transformation vector pRI 101-AN (TaKaRa Bio Inc., Dalian, China) containing the 35S promoter of cauliflower mosaic virus (CaMV). Sequences recognized by *Xba*I, *Nde*I were added in front of the 5′-end of the gene, and sequences recognized by *Sal*I, *Eco*RI, *Sac*I enzymes were added behind the 3′-end of the gene. Original positions recognizing by *Nde*I (position 800), *Xba*I (712), and *Eco*RI (550) inside the *EeDAcT* gene were eliminated, but the open reading frame was conserved. Chemically competent *Escherichia coli* cells, strain DH10B (New England Biolabs Inc., Ipswich, MA, USA), were used for transformation. Transformed bacteria were selected on LB medium [26] agar plates containing kanamycin and verified by molecular analysis of plasmid. The plasmid containing pRI-101AN-nat*EeDAcT* construct was used for transformation of *Agrobacterium tumefaciens* cells by electroporation [27]. Positive transformants were selected on LB medium agar plates containing kanamycin and rifampicin and cultivated in 10 mL liquid LB medium containing 10 μg/mL of rifampicin and 50 μg/mL of kanamycin, under shaking (250 rpm) at 28 °C for 24–36 h. An optical density (OD_600_) of 0.8–1.0 was obtained after 24–36 h, measured with NanoDrop 2000 spectrophotometer (Thermo Scientific, Waltham, MA, USA).

Leaf discs of tobacco were transformed using *Agrobacterium*-mediated protocol [28]. Kanamycin at concentration 50 μg/mL was used as a selection pressure during regeneration of transformed cells and rooting of regenerated shoots. Transgenic plants were transferred from in vitro to in vivo and cultivated in greenhouse conditions. 

Presence of the *EeDAcT* transgene was detected in transgenic tobacco plants by PCR using primer pair 5′-TCGCTCCCTTGAACATCTCT-3′ and 5′-GGAAAATAAGCCCAACGTGA-3′. Expected size of the PCR product was 579 bp. The PCR reaction mixture and thermocycler type were the same as previously. The PCR parameters were as follows: initial denaturation at 94 °C for 3 min, followed by 32 cycles, each consisting of a denaturation at 94 °C for 1 min, annealing at 60 °C for 25 s, extension at 72 °C for 1 min, and the final extension step at 72 °C for 10 min. PCR products were separated in 1.5% (*w*/*v*) agarose gel in 1xTBE buffer (1.1% Tris-HCl; 0.1% Na_2_EDTA; 0.55% boric acid) pre-stained with 0.10 µL/mL of ethidium bromide.

## 3. Results

### 3.1. AcetylTAGs in E. europaeus

**Figure 1:**
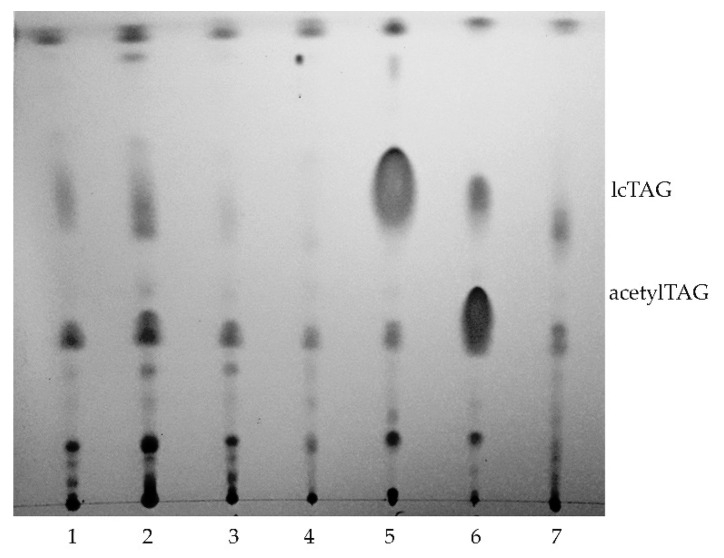
Detection of acetylTAGs (3-acetyl-1,2-diacyl-*sn*-glycerols) in different parts of *E. europaeus* (1—leaves, 2—stem, 3—flower, 4—pericarp, 5—aril, 6—immature seed, 7—root).

Presence of acetylTAGs was analyzed in root, stem, leaf, flower, aril, pericarp, and immature seeds of *E. europaeus*. Only immature seeds contained a high level of acetylTAGs. On the opposite, the aril tissue surrounding the seed produced the highest levels of long-chain triacylglycerols (lcTAGs). Other evaluated tissues and organs accumulated lcTAGs, but not acetylTAGs (Figure 1). 

**Figure 2:**
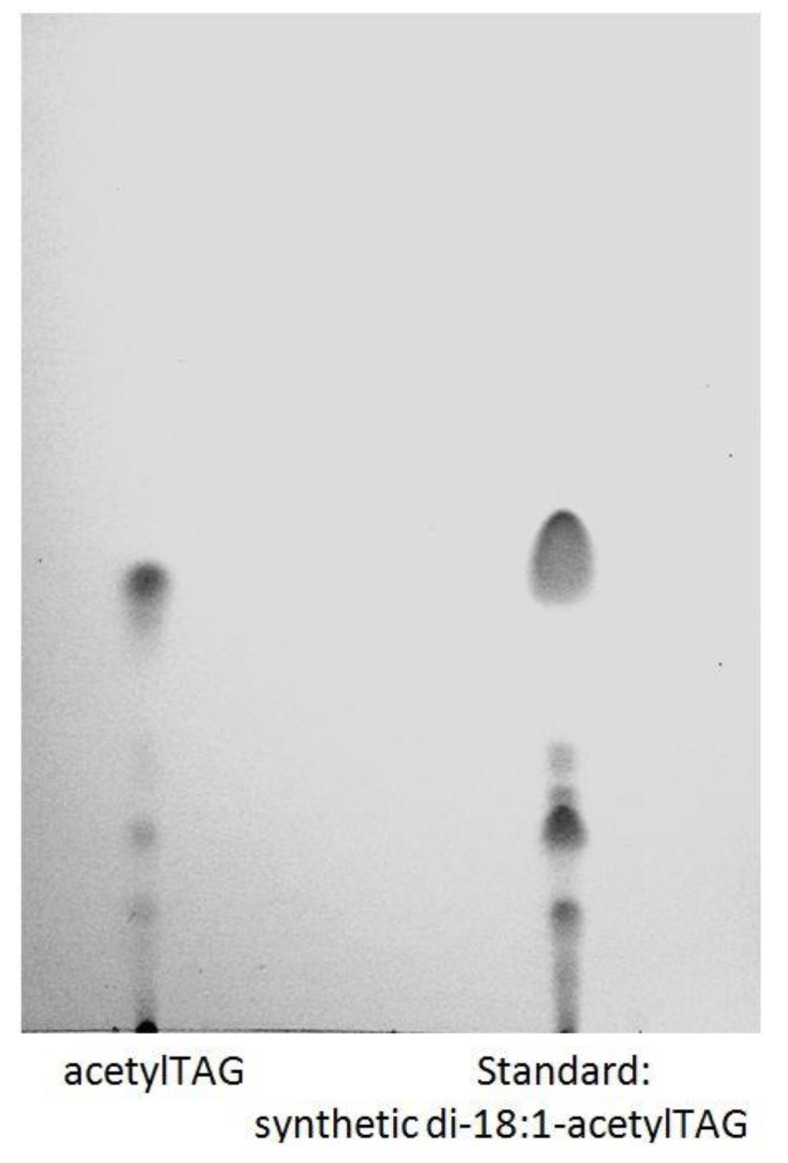
Comparison of acetylTAGs isolated from immature seeds of *E. europaeus* (left) with the synthetic standard di-18:1-acetylTAG (right) by TLC.

The spot corresponding with acetylTAGs from immature seeds of *E. europaeus* was scraped from the TLC plate, extracted, re-analysed, and compared with the standard of acetylTAGs, the synthetic di-18:1-acetylTAG. Analysis confirmed the presence of acetylTAGs in immature seeds of *E. europaeus* (Figure 2).

**Figure 3:**
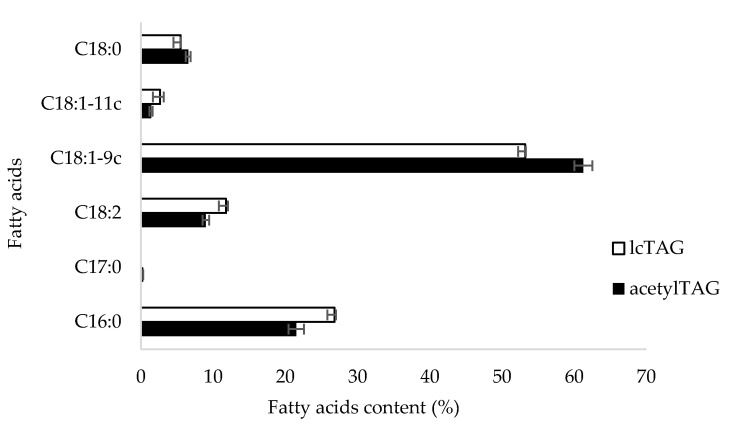
Relative fatty acids content (%) in acetylTAGs and lcTAGs (long-chain triacylglycerols) in oil of immature seeds of *E. europaeus* (C16:0—palmitic acid, C17:0—heptadecanoic acid, C18:2—linoleic acid, C18:1–9c—oleic acid, C18:1–11c—cis-vaccenic acid, C18:0—stearic acid).

The dominant fatty acid in acetylTAGs as well as in lcTAGs of *E. europaeus* immature seeds was the oleic acid (Figure 3). The fraction of acetylTAGs contained 61.3% and fraction of lcTAG 53.2% of oleic acid, respectively. Seeds accumulated also 9.0% of the essential linoleic acid in acetylTAGs and 11.8% in lcTAGs. The palmitic and stearic acids represented saturated fatty acids in acetylTAGs with a percentage 21.5% and 6.6%, respectively. Their content in lcTAGs was 26.8% and 5.5%, respectively. The cis-vaccenic acid was a minor component in both fractions of oil (1.4% and 2.7%, respectively), the heptadecanoic acid was not detected in lcTAGs and in acetylTAGs represented only 0.3%.

### 3.2. Diacylglycerol Acetyltransferase Gene from E. europaeus

**Figure 4:**
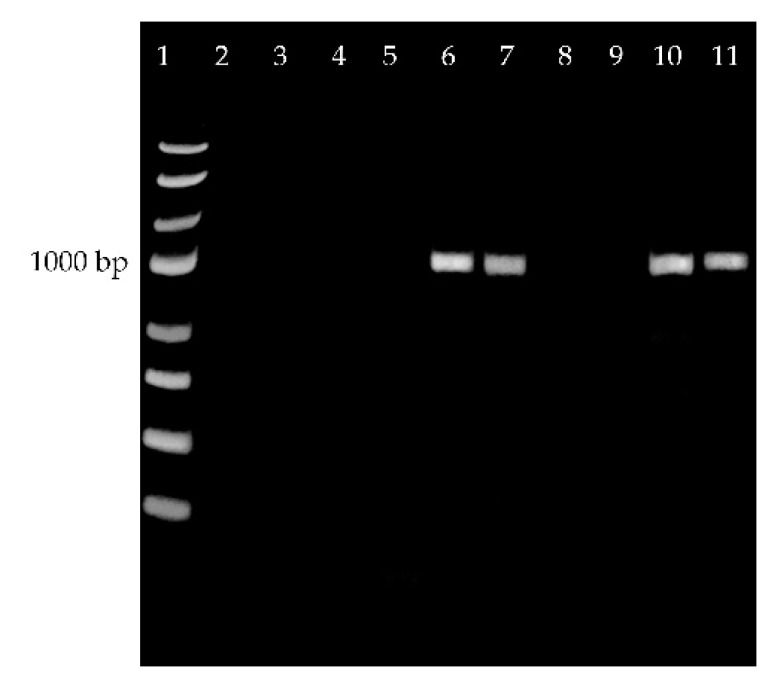
*EeDAcT* gene from *E. europaeus* amplified from cDNA (roots—lanes 2, stem—lane 3, leaves—lane 4, pericarp—lane 5, immature seeds—lanes 6, 7, aril—lanes 8, 9) and from genomic DNA (leaves—lanes 10, 11). Lane 1—100 bp DNA ladder.

Initial metabolomic analysis of lipids in seeds of *E. europaeus* confirmed the presence of 3-acetyl-1,2-diacylglycerols (acetylTAGs). Based on this prerequisite, the responsible diacylglycerol acetyltransferase-encoding gene was identified using high homology of this gene within the family Celastraceae Celestraceae. Primers for detection of *DAcT* in *E. europaeus* (*EeDAcT*) were designed according to available sequence of the *EaDAcT* gene from *E. alatus* (GenBank accession no. GU594061.1). Amplicons obtained by PCR analysis declared an expected length of gene of about 1092 bp (Figure 4, lanes 10, 11). Subsequently, a total RNA from individual organs and tissues (root, stem, leaf, aril, pericarp, immature seed) of *E. europaeus* was isolated and transcribed to cDNA. Primers derived from the *EaDAcT* gene of *E. alatus* provided positive amplifications and fragments of expected size only in immature seeds (Figure 4, lanes 6, 7).

**Figure 5:**
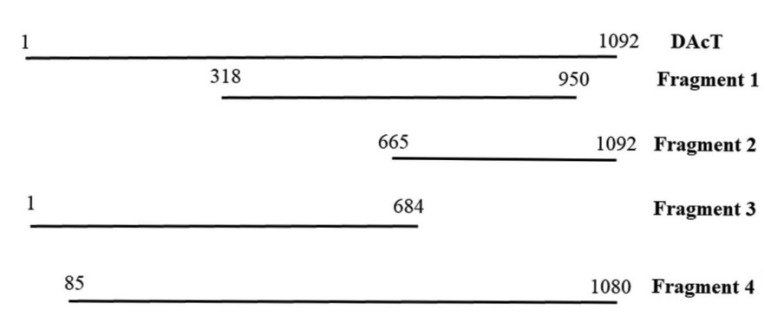
Overlapping of amplified cDNA fragments derived from natural *EeDAcT* gene.

Determination of cDNA sequence of *EeDAcT* gene was performed using a series of amplifications of cDNA and followed overlapping of amplified fragments (Figure 5). All fragments obtained from cDNA were sequenced, and the resulted nucleotide sequence of the natural *EeDAcT* gene has been submitted into the GenBank database (accession number MK637625.1).

**Figure 6:**
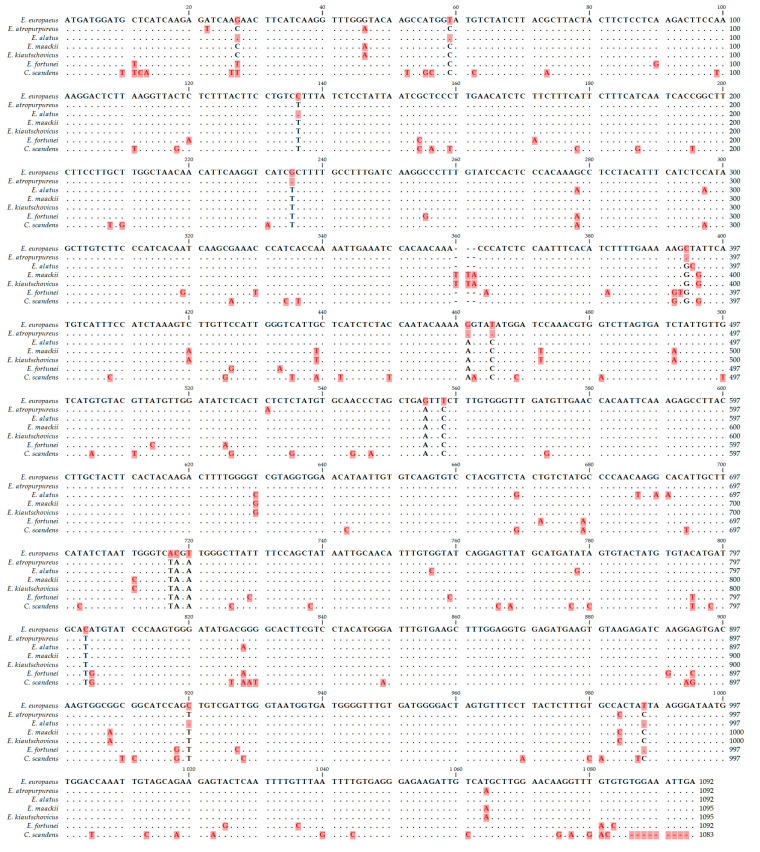
Alignment of cDNA sequence of *DAcT* genes of *E. europaeus* and other species within the family Celastraceae (red letters—differences against the *EeDAcT* gene, black letters—identical nucleotides).

**Figure 7:**
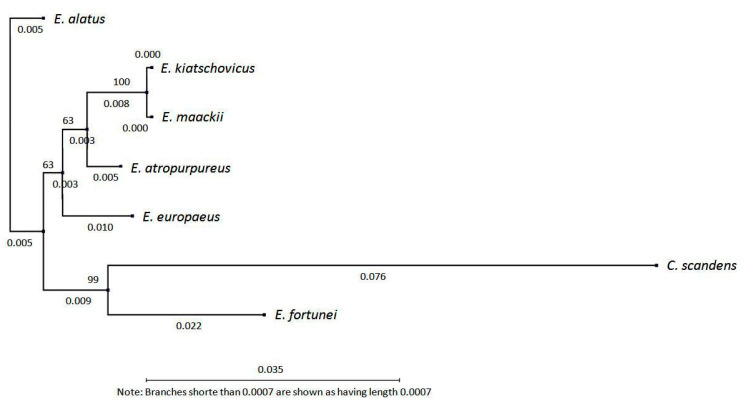
Phylogenetic tree of species of family Celastraceae Celestraceae according to cDNA sequences of *DAcT* genes. The branch length is proportional to the estimated divergence distance of each gene. The scale bar (0.035) corresponds to a 3.5% change. The percentage of replicate trees in which the associated taxa clustered together in the bootstrap test (100 replicates) is shown next to the branches.

Comparison of complete cDNA sequences of the *DAcT* genes with other species of the Celastraceae family declared the originality of the natural *EeDAcT* gene at six nucleotide positions (555, 558, 717, 718, 720, 804) (Figure 6). The highest degree of identity (98.53%) of cDNA sequence was of the natural *EeDAcT* gene with the homologous gene from *E. atropurpureus* (MF061249.1). High identity match was also observed with the homologous gene from *E. alatus* (GU594061.1). The lowest degree of similarity of *EeDAcT* was with the relevant cDNA sequence from *Celastrus scandens* L. (MF061248.1). This was also supported by the phylogenetic analysis (Figure 7). The phylogenetic tree created according to cDNA sequences of *DAcT* genes (Figure 7) resembled clustering of species of the genus *Euonymus* L. established by chemical composition of 3-acetyl-1,2-diacyl-*sn*-glycerols from seeds of mature fruits [10]. *E. europaeus* and *E. alatus* were separated into different clusters confirming their classification into different *Euonymus* subgenus. *E. alatus* belongs to the subgenus *Euonymus*, section *Melanocarya,* while *E. europaeus* belongs to subgenus *Kalonymus*, section *Kalonymus* [10]. However, it should be noted that neither our study nor the mentioned one may correlate with the current classic taxonomy of the genus *Euonymus* L.

**Figure 8:**
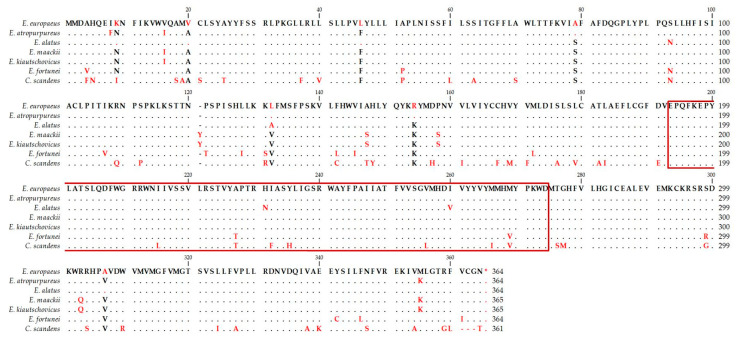
Alignment of amino acid composition of DAcT enzymes originated from *E. europaeus* and another six species of the family Celastraceae Celestraceae (red letters—differences against *EeDAcT* gene, black letters—identical amino acids).

The in silico analysis of amino acid composition confirmed the presence of a conserved domain of the O-acyl transferase family (MBOAT2) membrane-bound proteins at positions 193–274 (frame in the Figure 8). This was an important prerequisite for cloning the gene and its expression in transgenic plant tissues.

### 3.3. Expression of EeDAcT Gene in Tobacco 

Natural *EeDAcT* gene isolated from *E. europaeus* contained sequences recognized with the same restriction endonucleases that were used to clone this gene into a plasmid vector. Therefore, these sites were eliminated during the design process of synthetic *EeDAcT* gene. The open reading frame has not been changed, and changes in codon usage were minimized. Transformation vector pRI 101-AN contains constitutive promoter CaMV 35S and the 5′-untranslated region that should provide a higher expression of the gene of interest [29]. Resulted plasmid pRI 101-AN-*EeDAcT* was transformed into competent *Escherichia coli* cells DH10B and its sequence was verified by the Sanger sequencing. *Agrobacterium tumefaciens*, strain EHA105, was transformed with plasmid vector pRI 101-AN-*EeDAcT*, and transformed cells were selected using kanamycin (50 μg/mL) and rifampicin (10 μg/mL) and again verified by PCR analysis.

**Figure 9:**
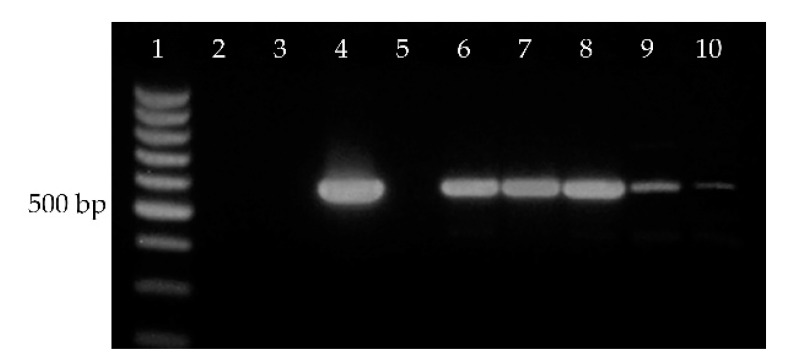
Presence of *EeDAcT* transgene in transformed tobacco plants (lane 1—100 bp DNA ladder, lanes 2, 3—negative control, lane 4—positive control (natural *EeDAcT* gene), lane 5—nontransgenic tobacco plant, lanes 6–10—transgenic tobacco plants T1–T5).

Presence of the *EeDAcT* transgene in transformed tobacco was confirmed by PCR analysis. Five regenerated and analyzed plants generated amplicons relevant to the presence of *EeDAcT* transgene (Figure 9, lanes 6–10).

**Figure 10:**
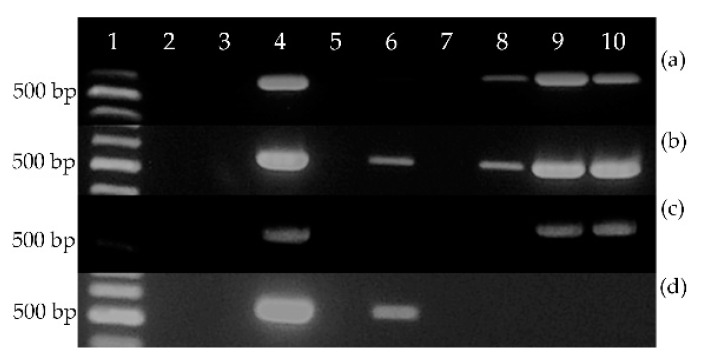
Analysis of *EeDAcT* gene expression in four different parts ((**a**) immature seeds, (**b**) stems, (**c**) leaves, (**d**) roots) of transgenic tobacco plants (lane 1–100 bp DNA ladder, lanes 2, 3—negative controls, lane 4—positive control, lane 5—nontransgenic tobacco plant, lanes 6–10—transgenic tobacco plants T1–T5).

Expression of the *EeDAcT* transgene was monitored using cDNA from individual parts of transgenic tobacco plants (root, stem, leaf, seed), and differences were detected (Figure 10). Transgenic plants T3, T4, and T5 expressed *EeDAcT* transgene in leaves, stems, and immature seeds, not in roots. The T1 transgenic plant expressed transgene in immature seeds, stems, and roots, not in leaves, and the T2 plant di not express the transgene in any organ. 

Analysis of *EeDAcT* transgene at the level of DNA as well as cDNA exhibited presence of PCR products with length 579 bp, corresponding with the expected length using designed primers (Figure 9 and Figure 10).

### 3.4. AcetylTAGs in Transgenic Tobacco 

**Figure 11:**
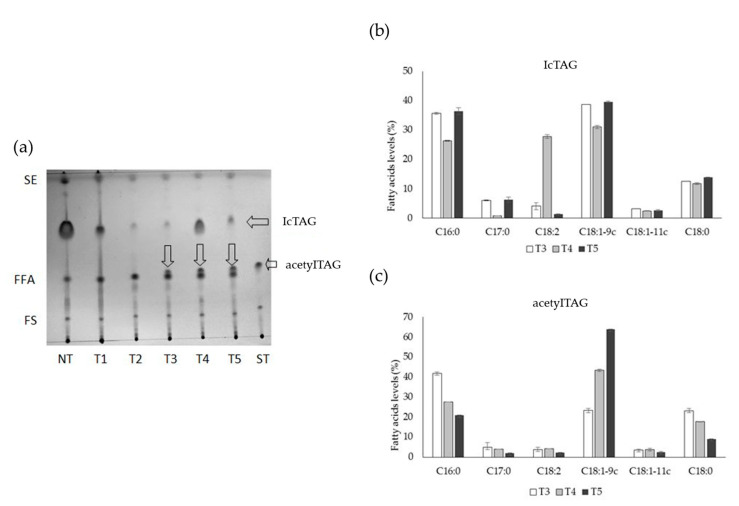
Accumulation of acetylTAGs in seeds of transgenic (T1–T5) and nontransgenic (NT) tobacco plants: (**a**) thin-layer chromatography (TLC) of lipid structures (FS—free sterols, FFA—free fatty acids, lcTAG—long chain triacylglycerols, SE—sterol esters); relative fatty acids content (%) in lcTAGs (**b**) and acetylTAGs (**c**) of transgenic tobacco plants T3, T4, T5 (C16:0—palmitic acid, C17:0—heptadecanoic acid, C18:2—linoleic acid, C18:1–9c—oleic acid, C18:1–11c—cis-vaccenic acid, C18:0—stearic acid).

The content of oil in immature seeds (approximately 12 days after pollination) of nontrangenic tobacco plant was 15.8% of fresh weight. Lipids (lcTAGs, acetylTAGs, free fatty acids (FFAs), free and esterified sterols, and polar lipids) were isolated and separated from immature seeds of transgenic and control tobacco plants by TLC coupled with densitometry. Nontransgenic control plants did not produce any acetylated TAGs, as they do not have the necessary genetic and thus no enzymatic background. New lipid structures were identified only in the three transgenic lines (T3, T4, T5). They were identified as a fraction of acetylTAGs, using the di-18:1-acetylTAG synthetic standard (Figure 11a). AcetylTAGs were the dominant lipids in immature seeds with content in the range of 29.5–54.8% and content of fatty acids in acetylTAGs was different in individual transgenic tobacco lines (Figure 11c). Lines were different also in the amount of accumulated lcTAGs as well as in content of fatty acids in lcTAGs (Figure 11b). T3 and T5 plants contained similar values (4.73% and 7.05%), but the T4 plant accumulated a very high content (53.9%) of lcTAGs. 

**Figure 12:**
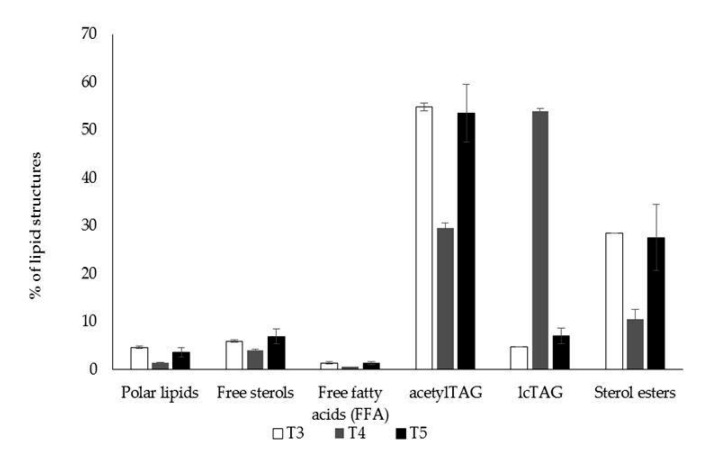
Accumulation (relative percentage) of acetylTAGs, lcTAGs, and other lipid structures in immature seeds of transgenic tobacco plants T3, T4, T5.

Polar lipids, free fatty acids, and free sterols were minor lipids in transgenic tobacco seeds (Figure 12).

**Figure 13:**
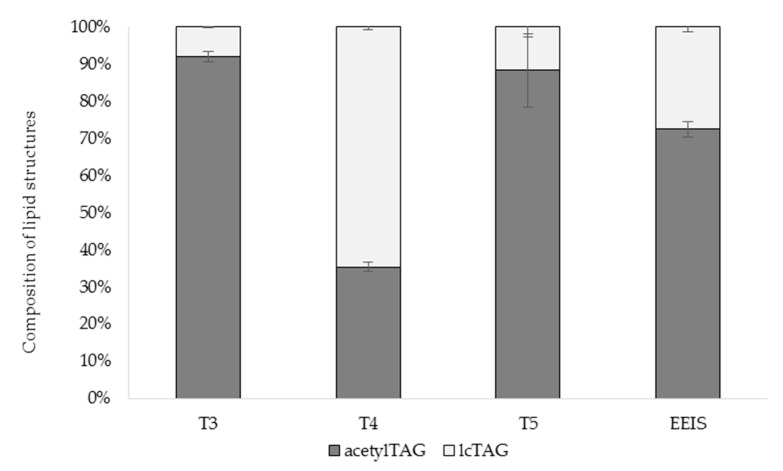
Relative ratios of acetylTAG and lcTAG contents in immature seeds of transgenic tobacco plants (T3, T4, T5) and *E. europaeus* (EEIS).

Proportion of acetylTAGs and lcTAGs in immature seeds of tobacco transformed with modified *EeDAcT* gene showed a strong effect of transgene (Figure 13). T3 and T5 plants synthesized a high level of acetylTAGs (54.8% for T3 and 53.5% for T5). lcTAGs were a minor compound, with content of 4.7% (T3) and 7.1% (T5). The T4 plant had the opposite parameters. It contained 29.5% of acetylTAGs, but 53.9% of lcTAGs. The proportion of acetyl TAGs to lcTAGs in two tobacco transgenic lines (T3, T5) was similar, even higher than in immature seeds of *E. europaeus* (Figure 13).

Oleic acid was the dominant fatty acid in acetylTAGs of all transgenic plants. However, its concentration markedly varied between transgenic samples (63.8% in T5 and 23.2% in T3 plant). The opposite trend was observed in the case of palmitic acid. Its level was the highest in T3 (41.9%), compared with T4 (27.5%) and T5 (20.8%) plants. Stearic acid was present in acetylTAGs with values lowest in T5 (8.9%), followed by T4 (17.6%) and T3 (23.1%) plants. Other fatty acids were in minority. 

The fatty acid composition in lcTAGs showed similarities in contents of oleic and palmitic acids, but differences in linoleic acid. T4 plant accumulated the lowest levels of palmitic (26.3%) and oleic acids (31.0%), but a significant higher level of linoleic acid (27.7%) compared with T5 (only 1.3%) and T3 (4.1%). 

## 4. Discussion

*Euonymus europaeus* L., similarly to other *Euonymus* species, possesses the genetic background and biochemical pathways for the synthesis of 3-acetyl-1,2-diacyl-*sn*-glycerols. It accumulates acetylTAGs in mature seeds and a very limited content also in arils [9]. The studies were performed almost exclusively on a related species, *E. alatus*. Immature seeds of *E. europaeus* used in this study also contained a high content of acetylTAGs, but they were not detected in aril tissues surrounding seeds (Figure 1 and Figure 3). As was previously published in regard to *E. alatus* [17,20], the *sn*-1 and *sn*-2 positions of acetylglycerides of *E. europaeus* are esterified with common fatty acids, predominantly with oleic acid, followed by palmitic and linoleic acids (Figure 3). Consequently, *E. europaeus* could be included among the candidates from the family of Celastraceae for isolation, cloning, and utilization of the gene encoding the responsible enzyme, diacylglycerol acetyltransferase. Compared with *EaDAcT*, the *EeDAcT* gene is unique. The enzyme *Ee*DAcT itself is different in six amino acids against *Ea*DAcT, and there are even greater differences compared with other *Euonymus* DAcTs (Figure 8). This suggests that the acetylation activity of *Ee*DAcT could be different from that of other *Euonymus* DAcTs. This assumption is based on an already revealed significant variation in activity of diacylglycerol acetyltransferases originated from other *Euonymus* species expressed in transgenic yeast [18].

The nucleotide sequence of cDNA obtained by reverse transcription from RNA isolated from immature seeds and the DNA sequence of the original *EeDAcT* gene isolated from *E. europaeus* were identical. This suggests that the *EeDAcT* gene has a simple, intronless structure. Such genes occupy approximately about one-fifth of all protein-encoding genes within plant genomes [30]. They include housekeeping genes, for example, genes encoding key enzymes included in the primary metabolism, storage proteins, and other proteins [31,32]. The fact that the *EeDAcT* gene does not contain introns emphasizes its importance in plant metabolism and plant life. This also increases the interest to transfer such genes into oil-producing plant species. Due to the intronless structure of the natural *EeDAcT* gene, only minor in silico redesigning and synthesis of an artificial gene for transformation into the host plant was necessary. Tobacco plants were used for heterologous expression of the *EeDAcT* gene. Although tobacco with a modified metabolic pathway of lipid biosynthesis can be also considered as a promising non-food crop for biofuel production [33], here it was used only as a model plant species for overexpression of isolated and modified *EeDAcT* gene. Results of metabolomic analysis in immature seeds of transgenic tobacco have coincided with the *EeDAcT* gene expression detected at the transcriptomic level. Expression of *EeDAcT* transgene varied within individual transgenic plants, and different levels of synthesized acetylTAGs in plants were associated with this. None of the transformed plants transcribed the *EeDAcT* gene in all four monitored parts (immature seeds, stems, leaves, roots) (Figure 10). There are several possible reasons, such as the positional effect of transgene, regulation sequences and flanking sequences of host DNA in the site of transgene integration, transgene copy number in the host genome, and epigenetic effects of gene silencing [34,35,36,37]. Therefore, the impact of the enzymatic activity of the transgene product was evaluated. That is the best indication of changes caused by the expression of alien gene in the metabolism of the host plant. 

The natural composition of fatty acids in tobacco seed oil is very diverse and depends on the tobacco genotype. Linoleic acid is dominant in some genotypes, the oleic acid in others, and in some genotypes there is a relatively well-balanced ratio between linoleic, oleic, and palmitic acids [38]. These ratios are more or less constant also in different vegetation conditions. Serious shifts in oil content and fatty acid composition tend to be associated with overexpression of alien genes related to lipid biosynthesis. This is also a typical impact of *DAcT* gene transfer into the host plants [33,39,40]. However, the main aim of introduction of *DAcT* gene is acetylation of synthesized TAGs. Transgenic tobacco plants obtained by introduction of *EeDAcT* gene generated both changes. They synthesized acetylated TAGs, but also variation in the content of individual fatty acids in acetylTAGs appeared. The palmitic acid dominated in acetylTAGs in T3 transgenic line, while oleic acid dominated in T4 and T5 lines (Figure 11b). There was also variation in the content of lcTAGs. Two lines (T3, T5) produced a very low amount of lcTAGs, whereas the line T4 produced predominantly lcTAGs (Figure 12). Differences in content of bounded fatty acids in lcTAGs were also found. High content of linoleic acid was present in lcTAGs in T4 transgenic line, very low in two others, but in the case of oleic acid it was vice versa. 

Summarizing of these results reveals that the presence of *EeDAcT* transgene, along with the impact of genetic transformation and transgene integration effect itself, causes extensive changes in lipid metabolism of the host plants. In addition to the acetylation of TAGs, changes also occur in the composition and relative proportions of lipid structures and fatty acids. Experiences from transgenic yeast and *Arabidopsis* seeds expressing the *Ea*DAcT from *E. alatus* revealed that this enzyme can acetylate a wide range of diacylglycerol substrates [16]. This can result in significant biochemical, physiological, and even morphological changes in the host organism that must be studied. This enzyme can also induce changes in the plant itself, giving the plant better resistance to environmental stresses. Overexpression of genes encoding different DGATs in a transgenic plant also changes quality parameters of oils required for either technical or nutritional use. Therefore, this approach could be extremely important for the development of new genotypes of plants advantageous for crop production. 

From the point of view of possible applications of *EeDAcT* gene isolated from *E. europeaus,* the most important conclusion is that the enzyme *Ee*DAcT encoded by this transgene was able to produce acetylate TAGs in transgenic plants. The host plants accumulated acetylTAGs not previously present, in a ratio similar to that in *E. europaeus*. 

## Figures and Tables

**Table 1:** Primer sequences for amplification of partial cDNA products.

Fragment	Primer Sequences (5′→3′)	PCR Product (bp)
1	F1 AATCAAGCGAAACCCATCAC R1 ATCACAAACCCCATCACCAT	632
2	F2 CGACTGTCTATGCCCCAACT R2 TCAATTTCCACACACAAA	427
3	F3 ATGATGGATGCTCATCAAGA R3 AGTTGGGGCATAGACAGTCG	684
4	F4 TCCTCAAGACTTCCAAAAGGA R4 CACAAACCTTGTTCCAAGCA	1007
